# You Can’t *B. cereus* – A Review of *Bacillus cereus* Strains That Cause Anthrax-Like Disease

**DOI:** 10.3389/fmicb.2020.01731

**Published:** 2020-08-19

**Authors:** Victoria M. Baldwin

**Affiliations:** Dstl, Salisbury, United Kingdom

**Keywords:** *Bacillus cereus*, *Bacillus anthracis*, anthrax, emerging disease, virulence plasmid

## Abstract

Emerging strains of *Bacillus cereus*, traditionally considered a self-limiting foodborne pathogen, have been associated with anthrax-like disease in mammals, including humans. The strains have emerged by divergent evolution and, as exchange of genetic material in the *Bacillus* genus occurs naturally, it is possible that further isolates will be identified in the future. The strains vary in their genotypes and phenotypes, combining traits of both *B. cereus* and *B. anthracis* species. Cases of anthrax-like disease associated with these strains result in similar symptoms and mortality rates as those caused by *B. anthracis.* The strains are susceptible to frontline antibiotics used in the treatment of anthrax and existing vaccines provide protection in animal models. The emergence of these strains has reignited the debate surrounding classification of the *B. cereus sensu lato* group and serves as a reminder that the field of medical microbiology is constantly changing and remains an important and ongoing area of research.

## Background

**FIGURE 1:**
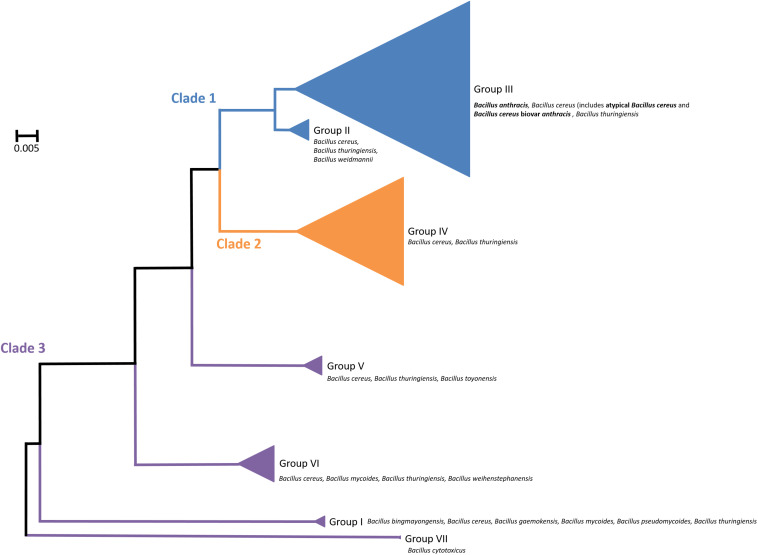
Organization of the *Bacillus cereus sensu lato* group, showing distribution of different strains in Clade 1 (blue), Clade 2 (orange) and Clade 3 (purple). These are further subdivided into seven groups. Triangle size is relative to the number of taxa analyzed per group. *Bacillus anthracis*, atypical *Bacillus cereus* and *Bacillus cereus* biovar *anthracis* are indicated in bold. Not shown are additional strains that are not assigned to a group.

*Bacillus cereus sensu lato* (*s. l.*) is a group of closely related Gram-positive, endospore-forming bacteria. Though genetically similar, these bacteria have diverse phenotypes with significant roles in agriculture, the environment, food spoilage and human and animal health. A pan-genome study of the group identified 59,989 different genes, of which 598 were considered “core” genes, defined as being present in 99% of the genomes analyzed. A total of 45% of the genes were unique to one strain within the group, which may be a contributing factor to the group’s diversity (Bazinet, 2017). The group is broadly divided into three clades, each containing strains of *Bacillus cereus sensu stricto* (hereafter, *B. cereus*) and *Bacillus thuringiensis* (Böhm et al., 2015; Okinaka and Keim, 2016; Bazinet, 2017; Fayad et al., 2019; Figure 1). In addition, Clade 1 contains all strains of *Bacillus anthracis* and Clade 3 is the most diverse, comprising of several other species; *Bacillus weihenstephanensis* (Lechner et al., 1998), *Bacillus mycoides* and *Bacillus pseudomycoides* (Nakamura, 1998), *Bacillus gaemokensis* (Jung et al., 2010), *Bacillus manliponensis* (Jung et al., 2011), *Bacillus cytotoxicus* (Guinebretière et al., 2013), *Bacillus toyonensis* (Jiménez et al., 2013), *Bacillus bingmayongensis* (Liu et al., 2014) and *Bacillus wiedmannii* (Miller et al., 2016). The clades are further divided into seven subgroups. Phylogenetic organization of the clades and subgroups is largely consistent, irrespective of the method used to define them. These include Bayesian statistics (Didelot et al., 2009) and phenotyping (for example, thermal tolerance) (Guinebretière et al., 2008). Genetic analyses include 16S rRNA gene sequencing (Lapidus et al., 2008), multilocus sequence typing (MLST) (Didelot et al., 2009), amplified fragment length polymorphisms (AFLP) (Guinebretière et al., 2008; Tourasse et al., 2010), whole genome sequencing of protein-coding genes (Schmidt et al., 2011; Zwick et al., 2012), single nucleotide polymorphisms (SNPs) (Liu et al., 2015), DNA-DNA hybridization (Böhm et al., 2015), pan-genome-wide association studies (Bazinet, 2017) and mobile genetic elements (Fayad et al., 2019).

There has been much debate surrounding the nomenclature of this group of bacteria. Recently, Carroll et al. (2020) proposed a novel taxonomy, reassigning the bacteria into the species *Bacillus pseudomycoides*, *Bacillus paramycoides*, *Bacillus mosaicus*, *Bacillus cereus sensu stricto*, *Bacillus toyonensis*, *Bacillus mycoides*, *Bacillus cytotoxicus* and *Bacillus luti.* Further division into subspecies would designate *B. anthracis* as *Bacillus mosaicus* subsp. *anthracis*. Whilst this method may aid clarity, it is yet to be seen whether it will be accepted by the wider scientific community. This is partly due to how deeply the current terminology is ingrained in published literature and day-to-day usage and partly because *B. anthracis* is a significant pathogen with severe consequences to human health and there is a great amount of legislation surrounding possession, use and transport of the bacteria and its products. Therefore, its importance is preserved with the distinction as a separate species.

Strains of *B. anthracis* are nested within Clade 1 of the *B. cereus s. l.* group and display little genetic variation (Keim et al., 2009). Differentiating between strains proved difficult until the development of variable number tandem repeat (VNTR) analysis (Jackson et al., 1997; Keim et al., 2000; Lista et al., 2006; Thierry et al., 2014). *B. anthracis* is a well-studied member of the group due to its role as a highly virulent obligate pathogen of mammals, including humans. Like other endospore-forming bacteria, *B. anthracis* exists in two states, the vegetative bacilli and a dormant spore. Sporulation occurs in the environment under nutrient-depleted conditions and spores are highly resistant to degradation by factors such as UV light, heat, desiccation, chemical disinfectants and antimicrobial compounds (Swick et al., 2016). Transmission to a host is usually via cutaneous, inhalational and gastrointestinal routes. Within the host, *B. anthracis* spores germinate and form vegetative bacteria that are capable of multiplying and producing virulence factors that may result in the potentially fatal disease, anthrax (see Moayeri et al., 2015 for a detailed review of anthrax pathogenesis). An unusual route of infection, via intravenous injection of heroin contaminated with *B. anthracis* spores, caused several deaths in the United Kingdom and wider EU (Brett et al., 2005; Grunow et al., 2012). Spores are able to persist in the environment for long periods of time. This was evidenced by the decades-long contamination of Gruinard Island in Scotland, which was exposed to aerosolised spores of virulent *B. anthracis* in 1942 for research purposes during World War II (Manchee et al., 1981). Viable spores were still recoverable in the 1980s, so extensive treatment of the land with formaldehyde was undertaken to reduce the number of spores to a safe level (determined by safely grazing sheep on the land for 5 months with no fatalities) (Manchee et al., 1994).

In contrast, *B. cereus* strains are spread throughout the *B. cereus s. l.* group, with pathogenic variations found in Clades 1 and 2. Contamination of food with pathogenic *B. cereus* is common and can cause spoilage and either emetic or diarrheal foodborne disease in humans, which is usually self-limiting (Wijnands et al., 2006; Fricker et al., 2007; Saleh-Lakha et al., 2017). However, virulence varies greatly, depending on the pathogen strain and host immune status (Chang et al., 2017). For example, *B. cereus* strain IP5832 can be included in probiotics for human consumption (Hoa et al., 2000), whilst other strains cause potentially fatal systemic food poisoning (Dierick et al., 2005; Naranjo et al., 2011; Public Health England, 2014). The two forms of disease are caused by the production of different toxins. Cereulide is associated with emetic symptoms (Agata et al., 2002; Häggblom et al., 2002) and enterotoxin with diarrhea (Granum and Lund, 1997; Senesi and Ghelardi, 2010). *B. cereus* is also able to form highly resistant spores that persist in the environment that are difficult to remove by traditional methods of decontamination (including cooking). *B. cereus* has several fundamental phenotypic differences when compared with *B. anthracis*. Unlike *B. anthracis*, it is typically hemolytic, motile, γ-phage resistant and penicillin G resistant (Kolstø et al., 2009).

Since 2004, reports of atypical *B. cereus* strains causing anthrax-like disease in humans and other mammals have emerged. These strains are defined by their *B. cereus* chromosomal DNA and the acquisition of virulence plasmids that are highly similar to the anthrax virulence plasmids pXO1 and pXO2.

**FIGURE 2:**
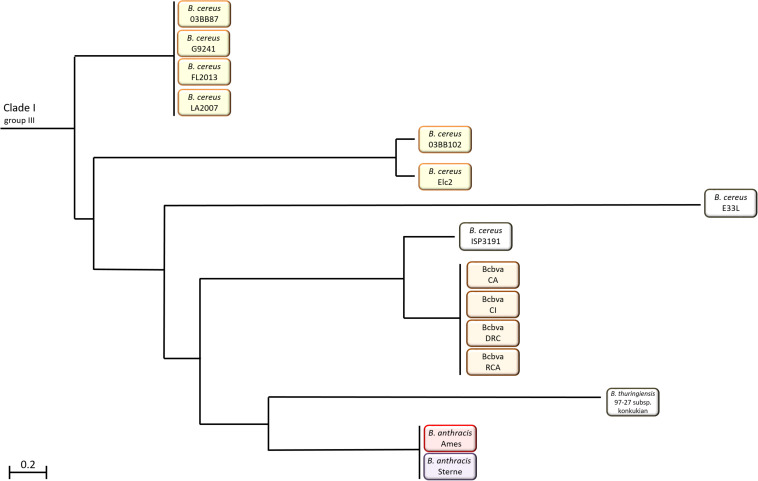
Distribution of atypical and Bcbva strains in Clade 1 of the *B. cereus sensu lato* group in relation to *B. anthracis* Ames.

Within the *B. cereus s. l.* group these strains appear in Clade 1 (Figure 1). Despite their ability to cause anthrax-like disease, they are more closely related to other *B. cereus* strains than *B. anthracis* (Antonation et al., 2016). There are two variants of *B. cereus* that cause anthrax disease; atypical strains such as G9241, FL2013 and 03BB102 and *B. cereus* biovar *anthracis* (Bcbva) strains such as CA and CI. The Bcbva variants are clustered together, derived from a single branch and their nearest neighbor is *B. cereus* strain ISP3191, which does not cause anthrax-like disease (Antonation et al., 2016). The atypical *B. cereus* strains can be found across different branches and are evolutionarily further from *B. anthracis* than Bcbva (Hoffmaster et al., 2006; Antonation et al., 2016). Other close neighbors to both variants include *B. cereus* E33L, known as Zebra-killer due to its isolation from a zebra carcass and *B. thuringiensis* 97-27 subsp. *konkukian* serotype H34, which was isolated from a human with a necrotic wound (Han et al., 2006; Hoffmaster et al., 2006; Klee et al., 2010; Antonation et al., 2016). Figure 2 shows the distribution of atypical and Bcbva strains in relation to *B. anthracis* Ames, based on the detailed phylogenetic trees published by Antonation et al. (2016) and Pena-Gonzalez et al. (2018).

In humans, isolated cases of pulmonary anthrax-like disease caused by atypical *B. cereus* were confirmed in metal workers from Louisiana and Texas (Hoffmaster et al., 2004, 2006; Avashia et al., 2007; Wright et al., 2011; Pena-Gonzalez et al., 2017). Of the seven total cases, six were ultimately fatal (Table 1). A mortality rate of 86% [based on a small number of cases (*n* = 7)] is consistent with that observed in pulmonary anthrax-like disease caused by *B. anthracis* (86–89%) (Kamal et al., 2011). There may have been earlier incidents of inhalational anthrax-like disease caused by atypical *B. cereus* strains; however, these were not verified at the time (Bekemeyer and Zimmerman, 1985; Miller et al., 1997). The infections and fatalities occurred in immunocompetent men with no known risk factors. However, they were all metal workers and may have been particularly at risk of infection via the inhalation route. Occupational hazards, including high numbers of spores in dust generated and damage to the respiratory tract, could increase their susceptibility to respiratory disease (Antonini et al., 2003). Additionally, two cases of cutaneous anthrax-like disease caused by *B. cereus* have been observed. The first incident occurred in a non-metal worker in Florida with an unknown cause of infection that resulted in development of a characteristic anthrax eschar (Marston et al., 2016). The second case was a lab-acquired infection of *B. cereus* G9241 in Illinois (Kaiser, 2011.). At least six different strains of atypical *B. cereus* (G9241, 03BB87 03BB102, Elc2, FL2013 LA2007, and LA4726) were responsible for these cases (Hoffmaster et al., 2004, 2006; Avashia et al., 2007; Wright et al., 2011; Marston et al., 2016; Pena-Gonzalez et al., 2017).

**TABLE 1:** Atypical and Bcbva strains discussed in this review including their place and source of origin, anthrax-like virulence plasmid content and disease caused.

Organism	Country of origin	Host/origin	Virulence plasmids	Symptoms	Outcome	References
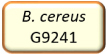	Louisiana, United States	Human; blood/sputum (welder, immunocompetent)	pBCXO1 pBC210	Fever, chills, dyspnea, hemoptysis, nausea, vomiting, cough, pneumonia	Pulmonary anthrax-like disease requiring intensive care Recovered	Hoffmaster et al., 2004, 2006
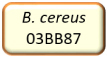	Texas, United States	Human; blood (muller, 56 years old, immunocompetent)	pBCXO1 pBC210	Chills, malaise, cough, hemoptysis, dyspnea, diarrhea, fever, pneumonia	Pulmonary anthrax requiring intensive hospital treatment Fatal	Hoffmaster et al., 2006; Avashia et al., 2007
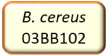	Texas, United States	Human; blood (welder, 39 years old, immunocompetent)	pBCXO1 pBC210	Abdominal pain, diarrhea, cough, fever, chills, vomiting, hypoxia, pneumonia	Pulmonary anthrax requiring intensive hospital treatment Fatal	Hoffmaster et al., 2006; Avashia et al., 2007; Pena-Gonzalez et al., 2018
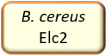	Texas, United States	Human; blood (welder, 39 years old, immunocompetent)	pBCXO1	Dyspnea, hemoptysis, vomiting, cough, headache, chest pain, pneumonia	Pulmonary anthrax requiring intensive hospital treatment Fatal	Wright et al., 2011; Pena-Gonzalez et al., 2018
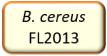	Florida, United States	Human; skin lesion swab (non-metal worker, 70 years old, immunocompetent)	pBCXO1	Black eschar skin lesion	Cutaneous anthrax requiring Antibiotics in hospital Recovered	Marston et al., 2016
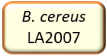	Louisiana, United States	Human; unknown sample (welder)	pBCXO1 pBC210	Pneumonia	Pulmonary anthrax Fatal	Pena-Gonzalez et al., 2017
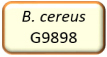	Louisiana, United States	Human; blood/sputum (two welders, 41 and 46 years old, immunocompetent)	pBCXO1 pBC210	Cough, chills, fever, hemoptysis, chest pain, pneumonia	Two cases of pulmonary anthrax requiring intensive hospital treatment. Both fatal	Miller et al., 1997; Sue et al., 2006
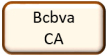	Dja Reserve, Cameroon	Apes (Chimpanzee and gorilla)	pBCXO1 pBCXO2	In chimpanzees; weakness, vomiting, sudden death	Often fatal	Leendertz et al., 2004; Klee et al., 2006; Klee et al., 2010
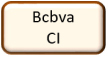	TaT National Park Cote d’lvoire	Chimpanzee	pBCXO1 pBCXO2	Weakness, vomiting, sudden death	Often fatal	Leendertz et al., 2004; Klee et al., 2006; Klee et al., 2010
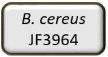	Koza, Cameroon	Bovine	pBCXO1 pBCXO2	High fever, potential bleeding from mucous membranes (e.g., nose), sudden death	Often fatal	Pilo et al., 2011
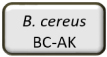	China	Kangaroo	pBCXO1 pBCXO2	Unknown	Unknown	Dupke et al., 2019

*Bcbva, Bacillus cereus biovar anthracis.*

Anthrax-like disease associated with *B. cereus* infection has also been identified in other mammals, including chimpanzees, gorillas, monkeys, elephants and various livestock, on large-scales across Western Africa (Leendertz et al., 2004, 2006; Klee et al., 2006; Pilo et al., 2011; Antonation et al., 2016; Hoffmann et al., 2017; Zimmermann et al., 2017). Most *B. cereus* strains associated with cases of anthrax-like disease in Africa are classified as Bcbva and are distinct from the atypical strains recovered from humans in the United States (Antonation et al., 2016; Hoffmann et al., 2017). However, an unusual strain originally designated *B. anthracis* JF3964, was isolated from cattle in Cameroon and is distinct from the closely related Bcbva strains despite possessing both pBCXO1 and pBCXO2 virulence plasmids (Tables 1, 3; Pilo et al., 2011; Antonation et al., 2016). Whilst no human infections with Bcbva have been observed, antibodies against Bcbva-specific antigen pXO2-60 have been detected in populations resident in the endemic Taï National Park region of Côte d’Ivoire (Dupke et al., 2020). In this region, a large proportion (38%) of wildlife mortalities are associated with anthrax-like disease caused by Bcbva (Hoffmann et al., 2017). Despite this, only 5% of wildlife was found to be seropositive for Bcbva. This low immune response may contribute to the high number of associated mortalities (Zimmermann et al., 2017).

Several studies have examined the virulence of atypical *B. cereus* G9241 in various mammalian models (Table 2). It has been shown to cause fatal anthrax-like disease in both immunocompromised and immunocompetent mice and in guinea-pigs, whilst one study demonstrated it is avirulent in New Zealand white rabbits (Wilson et al., 2011). Few studies have directly compared *B. cereus* G9241 with a strain of *B. anthracis* in the same experiment (Hoffmaster et al., 2004; Lever, unpublished data). These studies described similar levels of virulence between *B. cereus* G9241 and *B. anthracis* (Sterne and Ames respectively) (Table 2). However, many reports have included comparisons with previously published data, which generally suggests that *B. cereus* G9241 is less virulent than *B. anthracis* Ames and more virulent than *B. anthracis* Sterne (Oh et al., 2011; Wilson et al., 2011).

**TABLE 2:** *B. cereus* G9241 virulence in different mammalian models of infection.

Model	Route of infection	Dose (cfu)	LD_50_ (cfu)	Notes	Study
A/J mice^1^	Intraperitoneal	1 × 10^4^	Not measured	100% lethality at ∼116 h post inoculation Comparative challenge with *B. anthracis* Sterne: 100% lethality after ∼74 h post inoculation End of study: 5 days post inoculation	Hoffmaster et al., 2004
		1 × 10^6^	Not measured	100% lethality at ∼44 h post inoculation Comparative challenge with *B. anthracis* Sterne: 100% lethality at ∼52 h post inoculation End of study: 5 days post inoculation	
		1 × 10^2^–1 × 10^5^	381	100% lethality at doses of 10^4^ and 10^5^ cfu End of study: 14 days post inoculation	Oh et al., 2011
	Subcutaneous	10^3^–10^6^	1.3 × 10^3^	MTTD 3 days at 10^5^ cfu dose End of study: 14 days post inoculation	Wilson et al., 2011
			7	MTTD 3.5 days at 10^2^ cfu dose End of study: 14 days post inoculation	Scarff et al., 2016
			10	End of study: 14 days post inoculation	Seldina et al., 2018
	Intranasal	10^5^–10^7^	3.2 × 10^5^	MTTD 3 days at 10^6^ cfu dose End of study: 14 days post inoculation	Wilson et al., 2011
			3.0 × 10^3^	MTTD 4 days at 10^4^ cfu dose End of study: 14 days post inoculation	Scarff et al., 2016
			3.0 × 10^3^	End of study: 14 days post inoculation	Seldina et al., 2018
C57BL/6 mice^2^	Intraperitoneal	1.0 × 10^2^–1 × 10^5^	2,710	100% lethality at a dose of 10^5^ cfu End of study: 14 days post inoculation	Oh et al., 2011
		1.0 × 10^5^	Not measured	100% lethality within 4 days post inoculation End of study: 14 days post inoculation	Oh et al., 2013
			Not measured	MTTD 48.5 h End of study: 14 days post inoculation	Wang et al., 2013
	Subcutaneous	10^3^–10^6^	5.0 × 10^3^	MTTD 3 days at 10^5^ cfu dose End of study: 14 days post inoculation	Wilson et al., 2011
			44	MTTD 6 days at 10^2^ cfu dose End of study: 14 days post inoculation	Scarff et al., 2016
			40	End of study: 14 days post inoculation	Seldina et al., 2018
	Intranasal	10^5^–10^7^	6.3 × 10^5^	MTTD 3.5 days at 10^7^ cfu dose End of study: 14 days post inoculation	Wilson et al., 2011
			4.0 × 10^5^	MTTD 3 days at 10^7^ cfu dose End of study: 14 days post inoculation	Scarff et al., 2016
			4.0 × 10^5^	End of study: 14 days post inoculation	Seldina et al., 2018
	Aerosol	1.0 × 10^8^–2.0 × 10^10^	1.1 × 10^4^	100% lethality at dose of 2.3 × 10^5^ cfu End of study: 14 days post inoculation	Oh et al., 2013
Dunkin Hartley guinea-pigs	Aerosol	2.5 × 10^7^	Not measured	100% lethality in guinea-pigs immunized with a placebo within 5 days post inoculation End of study: 14 days post inoculation	Palmer et al., 2014
		4.1 × 10^2^–1.6 × 10^6^	Not measured	Median lethal dose 6,980 cfu compared to 10,100 cfu for *B. anthracis* Ames 100% lethality at doses of 10^5^ and 10^6^ cfu End of study: 15 days post inoculation	Lever, M. S. Dstl, unpublished data
New Zealand white rabbits	Subcutaneous	1.6 × 10^2^–1.6 × 10^5^	Not measured	Avirulent; 12/12 rabbits survived End of study: 14 days post inoculation	Wilson et al., 2011
	Aerosol	1.1 × 10^4^–1.1 × 10^7^	Not measured	6/7 rabbits survived with one mortality at 1.1 × 10^7^ cfu End of study: 14 days post inoculation	Wilson et al., 2011

*cfu, colony forming units; 1, inbred immunocompromised mice; 2, inbred immunocompetent mice; MTTD, median time to death.*

As with *B. cereus* G9241, experiments have shown that two Bcbva strains (CI and CA) are more virulent than *B. anthracis* Sterne but slightly less virulent than *B. anthracis* wild type strain 9602 (Brézillon et al., 2015). For example, the calculated LD_50_ values in outbred mice via the intranasal route were 3.5 × 10^4^ cfu Bcbva CI, 3.5 × 10^4^ cfu Bcbva CA, > 1.0 × 10^8^ cfu *B. anthracis* 7702 and 1.0 × 10^4^ cfu *B. anthracis* 9602 (Brézillon et al., 2015).

## Virulence Determinants

The emerging atypical *B. cereus* and Bcbva strains have obtained plasmids that enable expression of virulence factors to cause anthrax-like disease. These are highly related to the anthrax virulence plasmids pXO1 and pXO2. One of the traits that separate atypical *B. cereus* from Bcbva is that the atypical strains have obtained only one of these plasmids, pBCXO1, whereas Bcbva has obtained both pBCXO1 and pBCXO2. Several different combinations of chromosomal and plasmid DNA occur in *B. anthracis*, atypical *B. cereus* and Bcbva causing anthrax-like disease (Tables 3, 4). The essential requirement for full virulence is the expression of both tripartite anthrax toxin and a capsule (protein or polysaccharide).

**TABLE 3:**
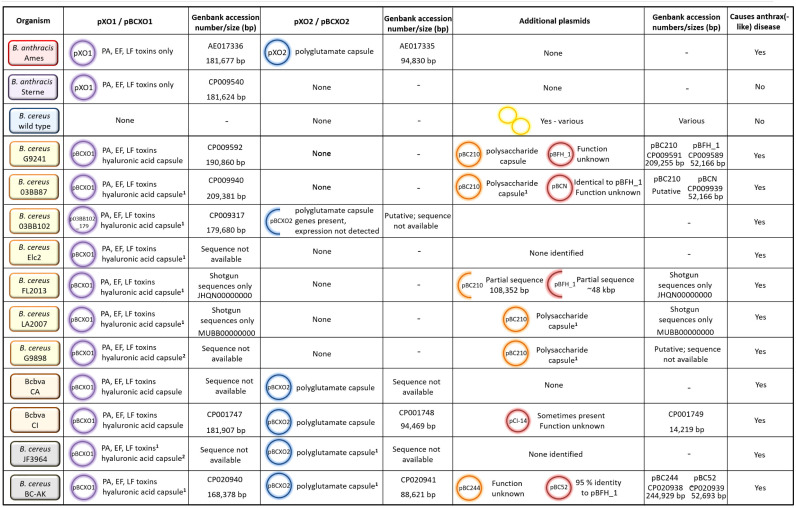
Plasmid possession, virulence factor expression and ability to cause anthrax-like disease for different strains of *B. anthracis* and *B. cereus.*

*Bcbva, *Bacillus cereus* biovar *anthracis*; 1, genes shown to be present by PCR or sequencing, expression unverified; 2, genes may or may not be present, sequence unknown.*

**TABLE 4:** Genetic and phenotypic differences between strains of *B. anthracis* and *B. cereus.*

Organism	AtxA regulator (anthrax toxins)	PIcR-PapR regulon (secondary factors)	*hasACB* operon (HA* capsule)	Hemolytic	Motile	y-phage resistant	Penicillin resistant	Causes anthrax (-like) disease	References
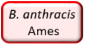	Functional	Nonsense mutation Non-functional	Frameshift mutation Non-functional	No	No	No	No	Yes	Read et al., 2003; Hoffmaster et al., 2006; Kolstø et al., 2009
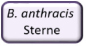	Functional	Nonsense mutation Non-functional	Frameshift mutation Non-functional	No	No	No	No	No	Cataldi et al., 2000; Kolstø et al., 2009
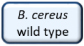	None	Functional	None	Yes	Yes	Yes	Yes	No	Kolstø et al., 2009
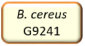	Functional	Functional	Functional	Yes	Yes	Yes	Yes	Yes	Hoffmaster et al., 2004, 2006
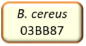	Functional	Functional	Functional	Yes	Yes	Yes	Not tested	Yes	Hoffmaster et al., 2006
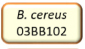	Functional	Functional	Functional	Yes	Yes	Yes	Not tested	Yes	Hoffmaster et al., 2006
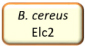	Functional	Functional	Functional	Yes	Yes	Not tested	Yes	Yes	Wright et al., 2011
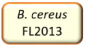	Functional	Functional	Functional	Yes	Not tested	Yes	Yes	Yes	Marston et al., 2016
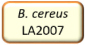	Functional	Functional	Functional	Not tested	Not tested	Not tested	Not tested	Yes	Pena-Gonzalez et al., 2017
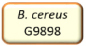	Functional	Sequence unavailable	Sequence unavailable	Yes	Not tested	Not tested	Yes	Yes	Miller et al., 1997; Sue et al., 2006
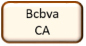	Functional	Frameshift mutation Non-functional	Functional	No	Yes	Yes	Yes	Yes	Klee et al., 2006; Antonation et al., 2016
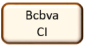	Functional	Frameshift mutation Non-functional	Functional	No	Yes	Yes	No	Yes	Klee et al., 2006; Antonation et al., 2016
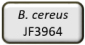	Functional	Sequence unavailable	Sequence unavailable	No	Not tested	Yes	Yes	Yes	Pilo et al., 2011
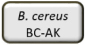	Functional	Functional	Functional	Not tested	Not tested	Not tested	Not tested	Yes	Dupke et al., 2019

*Bcbva, B. cereus biovar anthracis; Functional, genes of the operon do not contatin inactivating mutations, this does not indicate whether or not they are successfully expressed.*

### Anthrax Toxin

The anthrax toxin responsible for pathology and eventual fatality during the course of disease is a tripartite AB toxin comprised of protective antigen (PA), lethal factor (LF) and edema factor (EF). Expression of this toxin is essential for full virulence. The molecular mechanisms of anthrax toxin have been reviewed (Young and Collier, 2007; Moayeri and Leppla, 2009; Friebe et al., 2016). Briefly, PA binds to receptors on the surface of host cells and is cleaved by furin-like proteases. Truncated PA monomers assemble into heptamers and octamers, which embed into the cell membrane, creating a pre-pore formation. LF and EF then bind to the PA oligomers and the entire complex is endocytosed by the cell. The PA oligomer creates a pore in the endosome membrane, enabling release of LF and EF into the host cell cytoplasm. Here, LF functions as a Zn^2+^-dependent endoprotease, inhibiting mitogen-activated protein kinase kinase (MAP2K) activity, which disrupts cell signaling pathways and induces apoptosis (Klimpel et al., 1994). EF functions as a Ca^2+^- and calmodulin-dependent adenylate cyclase, increasing the concentration of cAMP inside the cell. This causes an osmotic imbalance, which interferes with cell signaling pathways and renders white blood cells ineffective but is not cytotoxic (Leppla, 1982).

In *B. anthracis*, the tripartite toxin is encoded by genes *pagA* (PA), *lef* (LF) and *cya* (EF) on the 181,677 bp plasmid, pXO1 (Okinaka et al., 1999). Bcbva strains harbor a similar sized plasmid, pBCXO1 (181,907 bp in the CI strain), which share 99–100% identity with pXO1 and encodes the genes for the toxins (Klee et al., 2010). In atypical strain G9241, the pBCXO1 plasmid is larger at 190,861 bp and shares 99.6% identity with pXO1 (Hoffmaster et al., 2004; Klee et al., 2010). Presumably, expression of these genes results in production of toxin components homologous to those found in *B. anthracis*, unless significant post-translational modification occurs. In one study, Marston et al. (2016) were able to detect LF, LF-neutralizing activity and anti-PA antibodies in the serum of a patient convalescing from cutaneous anthrax-like disease caused by an atypical *B. cereus* strain. Anti-LF and anti-PA Western blots also confirmed their presence in Bcbva strains (Brézillon et al., 2015). These data, coupled with the characteristic presentation of the disease [for example, formation of a black eschar (Marston et al., 2016)] suggests the anthrax toxins produced by *B. cereus* are not significantly different from those produced by *B. anthracis.*

### Extracellular Capsule

The second component required for full virulence is an extracellular capsule. In *B. anthracis*, a poly-γ-D-glutamic acid (polyglutamate) capsule is produced which prevents opsonization and phagocytosis of vulnerable vegetative bacilli (Scorpio et al., 2007, 2010).

As summarized in Table 3, there are several capsules that can potentially be expressed by atypical *B. cereus* and Bcbva strains dependent on the plasmids harbored. The first is a hyaluronic acid (HA) capsule, which may be expressed by atypical *B. cereus* and Bcbva strains. Like the anthrax tripartite toxin, genes encoding the HA capsule are harbored on the pXO1 (and pBCXO1) plasmid, encoded by the *hasACB* operon. In *B. anthracis*, the capsule is not expressed due to a frameshift mutation in *hasA*, which results in premature termination of translation (Okinaka et al., 1999). However, the pBCXO1 plasmid may possess a non-mutated *hasA* gene, enabling the HA capsule to be expressed. Functional genes for the *hasACB* operon have been identified in atypical *B. cereus* strains 03BB87, 03BB102, FL2013, LA2007, G9241, and Elc2 and in five Bcbva strains, including CA and CI (Pena-Gonzalez et al., 2018). Expression of this capsule was observed in atypical *B. cereus* strain G9241 and Bcbva strains CA and CI (Hoffmaster et al., 2004; Brézillon et al., 2015).

In addition to the HA capsule, several atypical strains are capable of producing a unique exopolysaccharide (Bps) capsule. It is encoded by a nine gene operon, *bpsX-H*, on plasmid pBC210 (formerly pBC218), which is not found in *B. anthracis* or Bcbva strains (Table 3). Homologs of the genes are found in other species, including *Streptococcus pyogenes*, allowing gene functions to be putatively assigned (Oh et al., 2011). Atypical *B. cereus* strains G9241, G9898, 03BB87, and LA2009 encode the Bps capsule and have been associated with fatal and non-fatal inhalational anthrax-like disease in humans (Miller et al., 1997; Hoffmaster et al., 2004; Sue et al., 2006). Additionally, strain FL2013 has a partial sequence for the pBC210 plasmid, but does not harbor the *bpsX-H* operon (Gee et al., 2014; Marston et al., 2016). The Bcbva strains do not possess the pBC210 plasmid; however, they do harbor the pBCXO2 plasmid that is highly similar to pXO2 from *B. anthracis* (Table 3). It encodes the *capBCA* genes, for expression of the polyglutamate capsule. This unusual proteinaceous capsule is required for full virulence in *B. anthracis*; for example, pXO2 is cured from the Sterne strain and is sufficiently attenuated in animals to be used as a live vaccine for livestock (Uchida et al., 1985; Cataldi et al., 2000). The Bcbva strains therefore, express the anthrax toxins and HA capsule from pBCXO1 and the polyglutamate capsule from pBCXO2. In addition to the Bcbva strains, one atypical strain isolated in the United States, *B. cereus* 03BB102, was found to possess the *cap* genes although there was no evidence to suggest the polyglutamate capsule is expressed (Hoffmaster et al., 2006). It is an unusual strain as it harbors partial sequences for pBCXO1 and pBCXO2 as well as additional plasmid pBC210 (Table 3; Hoffmaster et al., 2006; Pena-Gonzalez et al., 2018). Two further strains, *B. cereus* JF3964 and *B. cereus* BC-AK, isolated in China, also possess *cap* genes on a pBCXO2 plasmid (Pilo et al., 2011; Dupke et al., 2019; Table 3). However, these strains have not yet been shown to express the polyglutamate capsule.

Atypical *B. cereus* and Bcbva strains may express the HA capsule. When visualized by microscopy, the HA capsule can be observed forming a large protective layer around vegetative bacilli in strains of both atypical *B. cereus* and Bcbva (Brézillon et al., 2015; Scarff et al., 2018). In mouse models, for both atypical *B. cereus* and Bcbva strains, virulence was maintained with sole expression of the HA capsule via the inhalational route, with mild attenuation via the cutaneous route (Brézillon et al., 2015; Scarff et al., 2018). Atypical *B. cereus* strains with a missing or incomplete pBC210 plasmid have been associated with fatal inhalational anthrax-like disease (03BB102) and characteristic cutaneous anthrax-like disease (FL2013) in humans (Hoffmaster et al., 2006; Marston et al., 2016). These data suggest that encapsulation with HA alone (along with anthrax toxin expression) is sufficient to enable *B. cereus* to cause anthrax-like disease in mammals. Compared to the HA capsule, the Bps capsule is a less important virulence factor. When visualized by microscopy, the exopolysaccharide encapsulates the bacilli in a much thinner layer than the HA capsule (Oh et al., 2011; Scarff et al., 2018). In mouse models, deletion of the HA capsule from *B. cereus* G9241 resulted in an increased LD_50_ via subcutaneous and inhalational routes (Scarff et al., 2018) and increased time to death and reduction of mortality (Oh et al., 2011) despite production of the Bps capsule suggesting a level of attenuation. There are no known cases of anthrax-like disease in humans or other mammals caused by anthrax-toxin expressing *B. cereus* strains producing only the Bps capsule. In contrast, Bcbva strains with a deletion of only the HA capsule, retaining the polyglutamate capsule, caused no reduction in virulence (Brézillon et al., 2015).

### Certhrax Toxin and Other Virulence Factors

**FIGURE 3:**
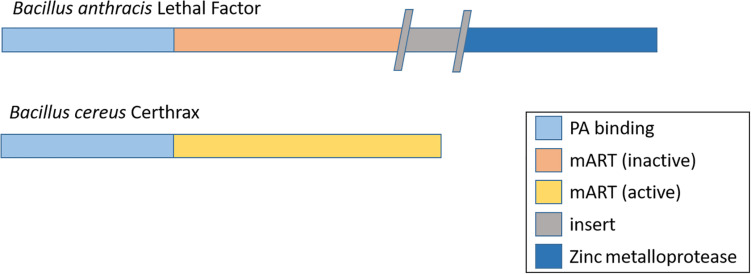
Domain organization of lethal factor from *B. anthracis* and certhrax from *B. cereus* G9241. Each contains a protective antigen (PA) binding domain and mono-ADP-ribosyltransferase (mART) domain. This is inactivated in lethal factor by an insertion. Lethal factor derives its toxicity from a metalloprotease domainwhich is absent in certhrax.

In addition to capsules and anthrax toxins, the emerging *B. cereus* strains possess other virulence factors not found in *B. anthracis*. For example, the pBC210 plasmid in *B. cereus* G9241 (and related atypical strains) encodes a mono-ADP-ribosyltransferase (mART) that has been designated certhrax toxin (Fieldhouse et al., 2010; Visschedyk et al., 2012; Simon et al., 2013; Simon and Barbieri, 2014; Seldina et al., 2018). It shares 51% structural similarity with *B. anthracis* LF. Each protein contains a PA binding domain that facilitates entry into the host cell. However, whilst the certhrax derives its toxicity from a mART domain, this is inactive in LF which possesses a functional metalloprotease domain (Figure 3; Visschedyk et al., 2012; Simon et al., 2013). Therefore, the two proteins cause toxicity via different mechanisms. The target for certhrax is vinculin which is part of the cytoskeletal complex and is involved in focal adhesion (Simon and Barbieri, 2014). Certhrax demonstrated 60x greater toxicity against RAW264.7 cells than LF (Simon and Barbieri, 2014). However, a recent study of LF, certhrax and LF/certhrax deletion mutants virulence in AJ and C57BL/6 mice demonstrated certhrax plays a minimal role in the virulence of *B. cereus* G9241 and may even cause attenuation (Seldina et al., 2018).

In addition to the certhrax toxin, atypical strains that possess the pBC210 plasmid also harbor PA and LF orthologs, designated protective antigen 2 (PA2) and CerADPr respectively (Oh et al., 2013; Seldina et al., 2018). The PA homolog in atypical *B. cereus* strains is highly similar to the PA in *B. anthracis*, with each domain sharing between 99 and 100% amino acid identity. In contrast, the PA2 domains share between 45 and 70% amino acid identity to PA from *B. anthracis* (Oh et al., 2013). Furthermore, PA2 is a weak virulence factor in mouse models compared to PA and is a poor antigen for immunization (Oh et al., 2013; Seldina et al., 2018). Whilst currently these virulence factors appear inconsequential, further structural or functional changes may enhance their significance as virulence factors in atypical *B. cereus* strains.

Another structural feature is the S-layer (or surface layer) which can play a role in virulence. In *B. cereus* G9241, many S-layer proteins share homology with those found in *B. anthracis* and its impairment can result in mild reduction in virulence (Wang et al., 2013).

Other virulence factors, such as hemolysis, motility and penicillin resistance are differentially expressed by atypical *B. cereus* and Bcbva strains depending upon genomic variation (see next section) (Table 4). Further genetic elements unique to different *B. cereus* strains may encode unidentified virulence factors. Functions for genomic islands I-VI in Bcbva strains, plasmid pCI-14 in Bcbva CI and pBFH_1 phagemid in *B. cereus* G9241 and related strains have not yet been elucidated (Table 3; Klee et al., 2010; Johnson et al., 2015; Antonation et al., 2016). *B. cereus* BC-AK also possesses an additional plasmid, pBC244, which appears unique to the strain and is of unknown function (Dupke et al., 2019; Table 3).

## Genetic Regulation

Two genomic elements in *B. anthracis* that regulate virulence factor expression are AtxA and the PlcR-PapR regulon. AtxA is a global regulator of virulence factors and its complex matrix of interactions has been reviewed (Fouet, 2010). Its best-known function is to upregulate the expression of tripartite anthrax toxin (PA, LF and EF). AtxA is active in *B. anthracis* strains and all *B. cereus* strains that cause anthrax-like disease, encoded on pXO1 and pBCXO1 respectively. In addition to the toxins, AtxA also upregulates the functional *hasACB* operon in atypical *B. cereus* and Bcbva strains for HA capsule expression (Brézillon et al., 2015; Scarff et al., 2016).

At least 45 genes are known to be under the control of the PlcR-PapR regulon, regulating a number of virulence factors such as enterotoxins, hemolysins and various proteases (Agaisse et al., 1999; Gohar et al., 2008). In *B. anthracis*, a nonsense mutation in the *plcR* gene disables the regulon and *B. anthracis* is typically non-hemolytic and does not produce enterotoxin (Agaisse et al., 1999; Mignot et al., 2001). It is proposed that the inactivated PlcR-PapR regulon and absence of accessory virulence factor expression contributes to the ability of *B. anthracis* to evade the mammalian immune system, establish an infection and ultimately cause disease. All other species in the *B. cereus s. l.* group, including *B. cereus*, possess a functional PlcR-PapR regulon. Within these species, approximately 1% of subspecies have a non-functional mutation (Slamti et al., 2004). Wild type *B. cereus* is therefore phenotypically distinct from *B. anthracis.*

In the atypical *B. cereus* strains, the PlcR-PapR regulon is active, enabling the expression of accessory virulence factors. Despite this, these strains are capable of causing anthrax-like disease. The mechanisms for this are poorly understood and are an area of ongoing research. A functioning PlcR-PapR regulon may also adversely affect sporulation efficacy; a study by Mignot et al. (2001) demonstrated a reduced ability for sporulation in *B. anthracis* with an activated PlcR-PapR regulon, suggesting conflict with a functioning gene for AtxA. However, this was contradicted by a later study, which showed rapid and complete sporulation is achievable in *B. anthracis* with an activated PlcR-PapR regulon (Sastalla et al., 2010). The reason for this discrepancy has not been elucidated and may be due to experimental differences (for example, the first study used homologous recombination to restore a functioning *plcR* gene on the chromosome, whereas the second study produced PlcR-PapR from a plasmid). However, there is evidence to suggest AtxA and PlcR are active under different growth conditions (Passalacqua et al., 2009). The full understanding of these inconsistencies and the precise mechanisms of both genetic regulatory systems could be an important area for future research. The atypical *B. cereus* strains also possess a second AtxA (designated AtxA2) on the pBC210 plasmid. It shares 79% identity with AtxA (Scarff et al., 2016). AtxA2 is capable of upregulating Bps capsule production and, to a lesser extent, HA capsule and tripartite toxin production. Deletion of AtxA2 results in a reduction in virulence in mouse models and deletion of both orthologs results in a mutant that is unable to sporulate (Scarff et al., 2016).

For Bcbva strains, the PlcR-PapR regulon has been inactivated by a frameshift mutation, which is different than the nonsense mutation in *B. anthracis* and has therefore evolved independently (Klee et al., 2010; Antonation et al., 2016). Phenotypically, the Bcbva strains are consistent with an inactive PlcR-PapR regulon (such as non-hemolytic and no phospholipase C activity).

Both atypical *B. cereus* and Bcbva strains are motile, whereas *B. anthracis* is characteristically immotile. This phenotype is caused by mutations in flagella genes that are functional in the *B. cereus* strains (Klee et al., 2010). One outlier is Bcbva strain DRC, which has an early stop codon in the *fliP* gene, rendering it immotile (Antonation et al., 2016). Whilst no motility genes were identified as under the control of the PlcR-PapR regulon by Gohar et al. (2008), an earlier study found PlcR binding sites in the promotor regions of some flagella genes (Ivanova et al., 2003). Further investigation is required to determine whether the PlcR-PapR regulon plays a role in the motility of these bacteria.

## Prevention and Treatment

Comprehensive advice on the prevention and treatment of anthrax can be found from the European Medicines Agency, Health Protection Agency and Centers for Disease Control and Prevention (European Medicines Agency/Committee for Human Medicinal Products (EMA/CHMP), 2014; Health Protection Agency [HPA], 2017; Centres for Disease Control and Prevention (CDC), 2015).

Any person thought to have been exposed to *B. anthracis* can be administered a post-exposure prophylactic course of oral antibiotics, usually ciprofloxacin or doxycycline. *B. cereus* is not known to be resistant to these antibiotics. Some strains of Bcbva showed intermediate sensitivity (mild resistance) to amoxicillin-clavulanic acid (Klee et al., 2006) and resistance to β-lactam antibiotics such as penicillin is commonly observed in *B. cereus* strains found in the food chain (Owusu-Kwarteng et al., 2017; Shawish and Tarabees, 2017). In severe cases of anthrax, or in patients with allergies to quinolones, ampicillin may be prescribed as a secondary drug. Therefore, correct identification of the causative bacteria could be important to ensure the most effective therapy is provided. However, only doxycycline and ciprofloxacin are licensed by FDA for use with inhalational anthrax (US Food and Drug Administration (FDA), 2008, 2016).

In the event of patients presenting with suspected anthrax, they will be treated according to the severity of their symptoms. Treatment could range from a course of oral antibiotics to intravenous antibiotic therapy, intensive care and surgery (for example, debridement in the case of injectional anthrax) as required.

### Vaccine

Vaccines produced in the United Kingdom and United States (anthrax vaccine precipitated (AVP) and anthrax vaccine adsorbed (AVA) respectively) largely induce an antibody response to the PA and, to a lesser extent, LF (AVP and AVA) and EF (AVA only). As the toxins expressed by atypical *B. anthracis* and Bcbva strains are homologous to those produced by *B. anthracis*, it is hypothesized that these currently licensed vaccines will provide adequate protection against anthrax-like disease caused by *B. cereus*. Studies in C57BL/6 mice and Dunkin Hartley guinea pigs confirmed that vaccination with PA is sufficient to provide protective, though not sterilizing, immunity against *B. cereus* G9241 (Oh et al., 2013; Palmer et al., 2014). Furthermore, a formaldehyde-inactivated spore and PA preparation generated immunity to Bcbva strains in outbred mice (Brézillon et al., 2015). Whilst there are no data from humans or primates and neither the United Kingdom nor United States vaccines are licensed for use against atypical *B. cereus* and Bcbva strains, it is likely both vaccines generate protective immunity against these emerging pathogens. However, this presumes there are no post-translational modifications or further evolution within the emerging strains and that pathology is not caused by other mechanisms. For example, whilst expression of certhrax toxin and PA2 has been demonstrated to be insufficient to generate full virulence (Seldina et al., 2018), further evolution may enable the atypical *B. cereus* strains to evade the vaccine. This highlights the need for continuing research and monitoring into emerging microbial pathogens.

### Anti-toxin

Anti-toxin antibodies may also be administered to a patient with inhalational anthrax. As with the vaccine, it is assumed that the anthrax toxins produced by *B. cereus* are homologous to those produced by *B. anthracis* and the treatment should be similarly effective. However, there is a wider debate ongoing as to whether anti-toxin therapy adds value to the treatment of anthrax disease (Vietri, 2018; Tournier et al., 2019).

## Summary

Classically, *B. anthracis* was considered the sole causative agent of anthrax disease in humans and mammals. However, in the past few decades, closely related strains of *B. cereus* have been identified that have obtained highly similar virulence plasmids and are capable of causing fatal anthrax-like disease. Two variants have emerged; atypical *B. cereus* strains that possess the pBCXO1 plasmid and Bcbva (*B. cereus* biovar *anthracis*) that possess both the pBCXO1 and pBCXO2 plasmids (Table 3). All of these strains produce the anthrax toxins and an extracellular capsule that enable them to cause anthrax-like disease. Atypical strains may produce a unique exopolysaccharide (Bps) capsule and Bcbva strains a polyglutamate capsule that is also expressed by *B. anthracis.* Additionally, both atypical and Bcbva strains may express a hyaluronic acid capsule that is encoded for but inactive in *B. anthracis* (Tables 3, 4).

Interestingly, the atypical strains have only been identified in the United States, whereas the Bcbva strains have been isolated in West African countries. Bcbva has caused widespread deaths in mammalian wildlife, including chimpanzees (Leendertz et al., 2004, 2006; Klee et al., 2006; Pilo et al., 2011; Antonation et al., 2016; Hoffmann et al., 2017; Zimmermann et al., 2017). To date, there have been no cases of anthrax-like disease recorded in humans caused by Bcbva. However, a recent study found serological evidence of human exposure to Bcbva in an endemic region of Côte d’Ivoire (Dupke et al., 2020). In contrast, the atypical *B. cereus* strains have been associated with fatal inhalational anthrax-like disease and characteristic cutaneous anthrax-like disease in humans. Despite all known human cases occurring in the United States, these incidents were separated geographically and temporally and involved several different strains, including those with the additional Bps capsule (such as G9241) and those without (such as FL2013) (Tables 1, 3; Hoffmaster et al., 2004, 2006; Avashia et al., 2007; Wright et al., 2011; Marston et al., 2016; Pena-Gonzalez et al., 2017). Therefore, it can be concluded that bacteria with a *B. cereus* chromosome are capable of causing anthrax-like disease if they are able to express the anthrax toxins and are encapsulated. There is also evidence for strains outside of these geographical areas; for example, Bcbva-like strain BC-AK was isolated from a kangaroo in China (Dupke et al., 2019). This suggests *B. cereus* capable of causing anthrax-like disease may already be distributed across the globe.

Fortunately, as the mechanism of pathogenicity is the same through production of tripartite anthrax toxin, it is highly likely that current anthrax vaccines will provide effective immunity against the atypical *B. cereus* and Bcbva strains (Oh et al., 2013; Palmer et al., 2014; Brézillon et al., 2015). The bacteria are also susceptible to frontline antibiotics, though administration of secondary β-lactamase antibiotics (such as penicillin) may have reduced efficacy due to inherent resistance in many *B. cereus* strains (Table 4; Klee et al., 2006).

Atypical *B. cereus* and Bcbva strains have diverse phenotypes and may retain other typical *B. cereus* characteristics such as motility, γ-phage resistance and production of secondary virulence factors (e.g., hemolysin) (Table 4). The apparent hybridity of these strains further demonstrates the close relatedness of the *B. cereus s. l.* group. The nomenclature applied to these strains is not well established and may be confusing. Here we propose “atypical strains” apply to those with a *B. cereus* chromosome and only the pBCXO1 plasmid, with “Bcbva” applied to those with a *B. cereus* chromosome and both the pBCXO1 and pBCXO2 plasmids. This could be extended to include *B. cereus* strains JF3964 and BC-AK as they also harbor both plasmids, despite not belonging to the cluster described by Antonation et al. (2016). It is also unclear how *B. cereus* 03BB102 should be defined, with its partial plasmids. It is evident that these strains are more diverse than previously thought and Carroll et al. (2020) have proposed a novel taxonomy for renaming the *B. cereus sensu lato* group to reflect the genomic and phenotypic variety. If it becomes widely accepted that the group consists of subspecies of the same species of bacteria, it could also be argued that, as the diseases associated with these *B. cereus* strains are caused by the production of anthrax toxin and manifest with classic anthrax symptoms, they should simply be called “anthrax” rather than “anthrax-like.”

To conclude, these *B. cereus* strains, traditionally considered foodborne pathogens that establish occasional opportunistic infections, have naturally evolved to cause fatal anthrax-like disease. This serves as a reminder that the field of medical microbiology is constantly changing, posing new challenges that require ongoing vigilance and research.

## Author Contributions

VB researched and wrote the article.

## Conflict of Interest

The author declares that the research was conducted in the absence of any commercial or financial relationships that could be construed as a potential conflict of interest.
